# Supplementary material for: Peripheral nerve induction of inhibitory brain circuits to treat Tourette syndrome: A randomized crossover trial
Source: medRxiv. 2023 Feb 6:2023.02.01.23285304. Preprint. [Version 2] doi: 10.1101/2023.02.01.23285304 (PMC9915823; doi:10.1101/2023.02.01.23285304)
Supplement: Supplement 2 [file media-2.pdf]

CONSORT 2010 checklist of information to include when reporting a randomised trial*

Section/Topic	Item No	Checklist item	Reported on page No
**Title and abstract**			
	1a	Identification as a randomised crossover trial in the title	Title page
	1b	Specify a crossover design and report all information outlined in table 2	Title and Abstract
**Introduction**			
Background and objectives	2a	Scientific background and explanation of rationale	Above Methods
	2b	Specific objectives or hypotheses	Just above Methods
**Methods**			
Trial design	3a	Rationale for a crossover design. Description of the design features including allocation ratio, especially the number and duration of periods, duration of washout period, and consideration of carry over effect	1st and 2nd par of Trial Design. Also Stimulation Protocol for design features.
	3b	Important changes to methods after trial commencement (such as eligibility criteria), with reasons	Par. Before Statistical Analysis.
Participants	4a	Eligibility criteria for participants	Participants, par 2
	4b	Settings and locations where the data were collected	Abstract: Setting
Interventions	5	The interventions with sufficient details to allow replication, including how and when they were actually administered	Stimulation protocol
Outcomes	6a	Completely defined pre-specified primary and secondary outcome measures, including how and when they were assessed	Outcome measures par 1-2
	6b	Any changes to trial outcomes after the trial commenced, with reasons	Outcome measures par 2.
Sample size	7a	How sample size was determined, accounting for within participant variability	Just above Trial Design
	7b	When applicable, explanation of any interim analyses and stopping guidelines	N/A
Randomisation:			
Sequence generation	8a	Method used to generate the random allocation sequence	Trial Design par 2
	8b	Type of randomisation; details of any restriction (such as blocking and block size)	Trial Design par 2

Allocation concealment mechanism	9	Mechanism used to implement the random allocation sequence (such as sequentially numbered containers), describing any steps taken to conceal the sequence until interventions were assigned	Trial Design par 2.
Implementation	10	Who generated the random allocation sequence,§ who enrolled participants, and who assigned participants to the sequence of interventions	Trial Design par 2.
Blinding	11a	If done, who was blinded after assignment to interventions (for example, participants, care providers, those assessing outcomes) and how	Trial Design par 2.
	11b	If relevant, description of the similarity of interventions	Trial Design par 2.
Statistical methods	12a	Statistical methods used to compare groups for primary and secondary outcomes which are appropriate for crossover design (that is, based on within participant comparison)	Statistical Analysis
	12b	Methods for additional analyses, such as subgroup analyses and adjusted analyses	Statistical Analysis
**Results**			
Participant flow (a diagram is strongly recommended)	13a	The numbers of participants who were randomly assigned, received intended treatment, and were analysed for the primary outcome, separately for each sequence and period	Figure 2 and Results par 1 and 2
	13b	No of participants excluded at each stage, with reasons, separately for each sequence and period	Figure 2
Recruitment	14a	Dates defining the periods of recruitment and follow-up	Methods, par 2 (subhead Participants)
	14b	Why the trial ended or was stopped	Methods, par 2 (subhead: Participants)
Baseline data	15	A table showing baseline demographic and clinical characteristics by sequence and period	Table 1
Numbers analysed	16	Number of participants (denominator) included in each analysis and whether the analysis was by original assigned groups	Fig. 2, 1st par. of Participants
Outcomes and estimation	17a	For each primary and secondary outcome, results including estimated effect size and its precision (such as 95% confidence interval) should be based on within participant comparisons.¶ In addition, results for each intervention in each period are recommended	Results section
	17b	For binary outcomes, presentation of both absolute and relative effect sizes is recommended	n/a
Ancillary analyses	18	Results of any other analyses performed, including subgroup analyses and adjusted analyses, distinguishing pre-specified from exploratory	"Outcome measures" and Results
Harms	19	Describe all important harms or untended effects in a way that accounts for the design (for specific guidance see CONSORT for harms)	Clinical Response and Harms
**Discussion**			
Limitations	20	Trial limitations, addressing sources of potential bias, imprecision, and, if relevant, multiplicity of	Limitations

		analyses. Consider potential carry over effects	
Generalisability	21	Generalisability (external validity, applicability) of the trial findings	Discussion / limitations
Interpretation	22	Interpretation consistent with results, balancing benefits and harms, and considering other relevant evidence	Discussion
**Other information**			
Registration	23	Registration number and name of trial registry	Abstract page
Protocol	24	Where the full trial protocol can be accessed, if available	Trial Design
Funding	25	Sources of funding and other support (such as supply of drugs), role of funders	Funding

CONSORT=Consolidated Standards of Reporting Trials.

- * Note: page numbers are optional depending on journal requirements.
- † Modified original CONSORT item.
- ‡ Unmodified CONSORT item.
- § Random sequence here refers to a list of random orders, typically generated through a computer program. This should not be confused with the sequence of interventions in a randomised crossover trial, for example receiving intervention A before B for an individual trial participant.
- ¶ A within participant comparison takes into account the correlation between measurements for each participant because they act as their own control, therefore measurements are not independent.

*We strongly recommend reading this statement in conjunction with the CONSORT 2010 Explanation and Elaboration for important clarifications on all the items. If relevant, we also recommend reading CONSORT extensions for cluster randomised trials, non-inferiority and equivalence trials, non-pharmacological treatments, herbal interventions, and pragmatic trials. Additional extensions are forthcoming: for those and for up to date references relevant to this checklist, see www.consort-statement.org.

Table 2 - Items to include when reporting a randomised crossover trial in a journal abstract

Item	Description
Authors	Contact details for the corresponding author
Trial design	Description of the trial design (crossover trial and number of periods)
Methods:	
Participants	Eligibility criteria for participants and the settings where the data were collected
Interventions	Interventions intended for all participants
Objective	Specific objective or hypothesis
Outcome	Clearly defined primary outcome for this report
Randomisation	How participants were allocated to sequences
Blinding (masking)	Whether or not participants, care givers, and those assessing the outcomes were blinded to group assignment
Results:	
Numbers randomised	Number of participants randomised to each sequence
Recruitment	Trial status
Numbers analysed	Number of participants analysed
Outcome	For the primary outcome, the estimated effect size and its precision based on within participant comparisons
Harms	Important adverse events or side effects
Conclusions	General interpretation of the results
Trial registration	Registration number and name of trial register
Funding	Source of funding
